# Clinical Outcomes in Routine Evaluation Measures for Patients Discharged from Acute Psychiatric Care: Four-Arm Peer and Text Messaging Support Controlled Observational Study

**DOI:** 10.3390/ijerph19073798

**Published:** 2022-03-23

**Authors:** Reham Shalaby, Pamela Spurvey, Michelle Knox, Rebecca Rathwell, Wesley Vuong, Shireen Surood, Liana Urichuk, Mark Snaterse, Andrew J. Greenshaw, Xin-Min Li, Vincent I. O. Agyapong

**Affiliations:** 1 Department of Psychiatry, Faculty of Medicine and Dentistry, University of Alberta, Edmonton, AB T6G 2B7, Canada; rshalaby@ualberta.ca (R.S.); andy.greenshaw@ualberta.ca (A.J.G.); xinmin@ualberta.ca (X.-M.L.); 2 Alberta Health Services Addiction and Mental Health, Edmonton, AB T5J 3E4, Canada; pamela.spurvey@albertahealthservices.ca (P.S.); michelle.knox@albertahealthservices.ca (M.K.); rebecca.rathwell2@albertahealthservices.ca (R.R.); wesley.vuong@albertahealthservices.ca (W.V.); shireen.surood@albertahealthservices.ca (S.S.); liana.urichuk@albertahealthservices.ca (L.U.); mark.snaterse@albertahealthservices.ca (M.S.); 3 Department of Psychiatry, Faculty of Medicine, Dalhousie University, Halifax, NS B3H 2E2, Canada

**Keywords:** CORE-OM, peer support worker, text messages, support, distress, hospital discharge, mental health, acute care

## Abstract

Background: Peer support workers (PSW) and text messaging services (TxM) are supportive health services that are frequently examined in the field of mental health. Both interventions have positive outcomes, with TxM demonstrating clinical and economic effectiveness and PSW showing its utility within the recovery-oriented model. Objective: To evaluate the effectiveness of PSW and TxM in reducing psychological distress of recently discharged patients receiving psychiatric care. Methods: This is a prospective, rater-blinded, pilot-controlled observational study consisting of 181 patients discharged from acute psychiatric care. Patients were randomized into one of four conditions: daily supportive text messages only, peer support only, peer support plus daily text messages, or treatment as usual. Clinical Outcomes in Routine Evaluation—Outcome Measure (CORE-OM), a standardized measure of mental distress, was administered at four time points: baseline, six weeks, three months, and six months. MANCOVA was used to assess the impact of the interventions on participants’ scores on four CORE-OM subscales across the three follow-up time points. Recovery, clinical change, and reliable change in CORE-OM all-item analysis were examined across the four groups, and the prevalence of risk symptoms was measured. Results: A total of 63 patients completed assessments at each time point. The interaction between PSW and TxM was predictive of differences in scores on the CORE-OM functioning subscale with a medium effect size (*F*_1,63_ = 4.19; *p* = 0.045; ηp_2_ = 0.07). The PSW + TxM group consistently achieved higher rates of recovery and clinical and reliable improvement compared to the other study groups. Additionally, the text message group and the PSW + TxM group significantly reduced the prevalence of risk of self/other harm symptoms after six months of intervention, with 27.59% (χ^2^_(1)_ = 4.42, *p* = 0.04) and 50% (χ^2^_(1)_ = 9.03, *p* < 0.01) prevalence reduction, respectively. Conclusions: The combination of peer support and supportive text messaging is an impactful intervention with positive clinical outcomes for acute care patients. Adding the two interventions into routine psychiatric care for patients after discharge is highly recommended.

## 1. Background

Psychological distress is often reported by patients who are discharged from a clinical setting [1,2,3,4,5,6]. Patients with mental health disorders are particularly vulnerable for suicidality or homicidal propensity [5,7,8]. Clinical Outcomes in Routine Evaluation—Outcome Measure (CORE-OM) is a well-recognized and validated tool that measures psychological distress and can be administered at several time points to detect changes in mental health parameters [9,10]. The instrument is designed to provide a feasible outcome measure. It is accepted by researchers and practitioners and sensitive to a wide range of intensities of psychological distress [10,11]. CORE-OM validity is supported by results of frequent trials and feedback received from practitioners [10].

A lack of routine follow-up care provided after clients are discharged from acute care units may lead to detrimental effects, including readmission and frequent emergency visits [12,13]. An estimated 12–22% of discharges from mental health hospitalizations may result in subsequent emergency department (ED) visits and 5–50% in hospital readmission depending on age and mental health conditions [14,15,16,17,18]. Several factors, including prior psychiatric hospitalization, multiple comorbidities, unemployment, and psychosis, have been identified as likely primary drivers of readmission within four years after hospital discharge [12,19].

Available peer support and texting services are interventions that can be easily integrated into health services given that each intervention has a proven track record of clinical effectiveness and economic value in mental health settings [20,21,22,23,24]. Improved mood status, sense of control of depression, and improved alcohol-use profile are associated with the use of supportive text messages for three or six months [24,25,26]. Similarly, lower rates of readmission within a supported recovery-oriented model have been observed with peer support services [20,27,28]. 

Providing these services to recently discharged patients may help alleviate potential health distress and close the treatment gap experienced by many after discharge, especially during the period preceding their first follow-up appointment with a healthcare provider, which may take up to weeks or months following discharge [29]. 

Monitoring changes in patients’ psychological parameters over time is of key importance for assessing the success and effectiveness of mental health services [9,30]. 

Roe et al. emphasized the importance of assessing patient outcome measures with active engagement of patients and service users who can participate in the selection and prioritization of the used measures [31]. Additionally, the available prevalent technologies, such as apps, can facilitate this involvement as they carry the promise of allowing easy collection, analysis, and presentation of ecologically valid patient-dependent outcome measures [31]. Recent research has found that person-centered and self-directed care approaches, among others, may allow individuals to exercise better control over their own care with benefits over usual care. These benefits are represented in rates of adherence and self-management as well as in medical and mental health outcomes, resulting in reduced inpatient and emergency room use and improved cost-effectiveness, service satisfaction, and quality of life [32]. These benefits may intensify when interventions become comprehensive, intensive, and integrated into routine care [32].

In this context, we undertook the current study to assess the effectiveness of supportive text messaging and peer support services using the CORE-OM as a window on intensity of psychological distress in patients discharged from acute care psychiatric units.

**Study objectives:** This study aimed to assess changes in overall CORE-OM and subscale scores for supportive text messaging and peer support treatment conditions compared to usual care across four study time points. 

## 2. Methods

The participants and study design were described in detail in a previous report [33]. A brief summary follows below.

### 2.1. Study Design

**Figure 1:**
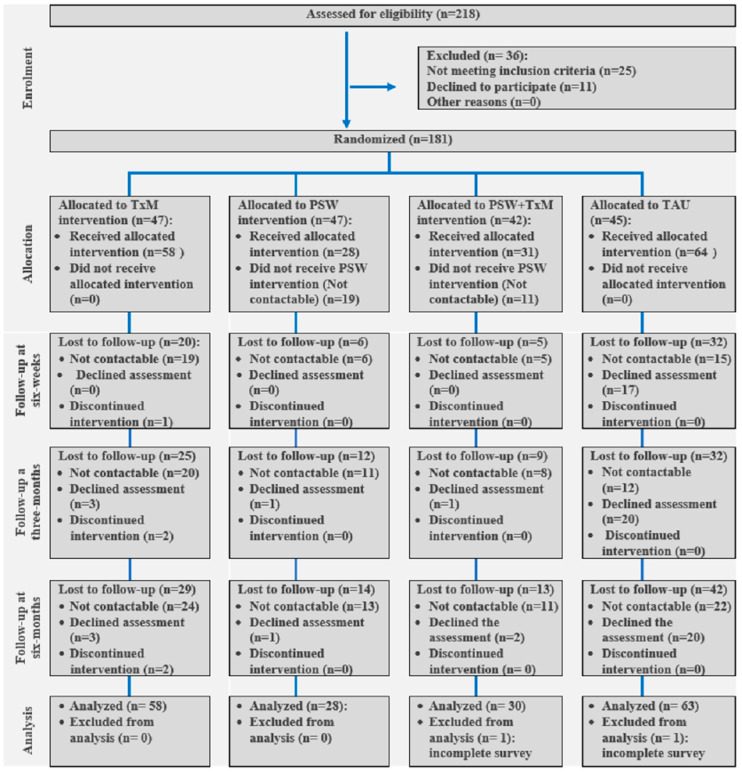
Study flow chart.

This controlled observational study was designed as a four-parallel-arm study with randomized services provided [29]. Although the initial intention was to conduct a randomized controlled trial (RCT), subject recruitment and treatment arm allocation issues necessitated an early planned transition to a controlled observational study, as described in the following sections. Participants were randomized into one of four conditions: (1) Peer Support Worker (PSW) only, (2) Text messaging services (TxM) only, (3) PSW plus TxM condition (PSW + TxM), and (4) treatment as usual (TAU), with 1, 1, 1, 1 allocation. Recruitment occurred from June 2019 to February 2020 from acute care units in Edmonton, Canada. A study flow chart is given in Figure 1. Researchers running follow-up assessment were blinded, and participants were allocated to treatment groups by computer-driven block randomization.

The study received ethics approval from the Health Ethics Research Board of the University of Alberta (reference number Pro00078427) and operational approval from Alberta Health Services. All participants provided written informed consent. The study is registered with ClinicalTrials.gov (trial registration: NCT03404882). Amendments to the study protocol [29] have been revised and registered (trial registration: NCT03404882).

Some patients who were randomized to receive the PSW intervention with or without TxM support did not receive PSW interventions for some reasons, such as subsequent noninterest in receiving visits from PSW, hospital readmission, and failure to reach out, despite several attempts being made to connect these patients with a PSW (Figure 1). Some of these participants continued to receive only TxM support or otherwise did not receive any service (acting like TAU group) and attended the follow-up assessment sessions related to the scale under study. Given the relatively small sample size of our study and the overarching objective of assessing the actual effects of the interventions, we adapted our study analysis plan to simply assess outcome data with regard to either TAU or TxM support-only groups to reflect the service the patients had actually received.

### 2.2. Patients

The inclusion criteria were as follows: (1) mental health condition (mood or psychotic disorder), (2) imminent discharge from acute care, (3) 18–65 years of age, (4) able to provide written consent, and (5) having a mobile handset capable of receiving text messages. The exclusion criteria were as follows: (1) inability to read text messages from a mobile device, (2) an addiction disorder without a mental health diagnosis, (3) receiving PSW service before the study, or (4) inability to commit to a sixth-month follow-up of the study.

### 2.3. Treatment Interventions

In the PSW-only condition, a PSW met the patients physically or virtually up to eight times over a six-month period to offer mental health support. In the TxM-only condition, only daily TxM were received. In the PSW + TxM condition, participants received both PSW services and daily TxM. In the control arm (i.e., TAU), only conventional follow-up appointments with community providers were offered.

Text4Support is a daily supportive text message service conceived and designed on cognitive behavioral therapy principles [33]. A bank of messages was generated and included message for the following eight mental health conditions: depression, anxiety, psychotic disorders, substance use disorders, bipolar disorder, adjustment disorders, attention-deficit or hyperactivity disorder, and general well-being. Examples of the messages are referred to in another publication [33].

### 2.4. Outcome

For this study, the primary outcome measure was psychological distress as assessed by the CORE-OM scale.

#### CORE-OM

CORE-OM is a 34-item self-report questionnaire designed for use as a baseline and outcome measure in psychological therapies. It consists of four main domains/items:

1. Subjective well-being (4 items, e.g., “I have felt overwhelmed by my problems”);
2. Problems/symptoms (12 items, e.g., “I have had difficulty getting to sleep or staying asleep”);
3. Functioning (12 items; e.g., “I have been able to do most things I needed to”);
4. Risk to self or others (6 items; e.g., “I have threatened or intimidated another person) [10,34].

Each item is rated on a Likert scale of five responses ranging from 0 to 4 (0 = not at all, 4 = always or most of the time). The lower the score, the better the psychological condition [35].

Three types of changes are usually studied with CORE-OM:

1. **Clinical change:** From the literature, clinical and nonclinical conditions usually relate to people who are waiting for therapy versus those who are not [36]. The cut-off score used is 10, and the change between two populations is called “clinical change” [36,37].
2. **Reliable change:** The reliable change index (RCI) is used to assess pre–post change, and 0.50 (clinical score of 5) is the cut-off score [10], i.e., clinical score changes greater than 5 are significant.

**Reliable improvement** indicates a positive reliable change, i.e., clients achieving a lowering of severity level by ≥5 [10,36,38]. Clients who have changes in score below +5 or −5 are deemed to have no reliable change, while clients with score increases of more than 5 are deemed to have deteriorated [10,38].

3. **Recovery:** Recovery is defined by two change conditions (clinical and reliable, as above) when baseline CORE-OM scores move from above the clinical range (equal to or greater than 10.0) to the nonclinical range and are reliably improved (change in score by ≥5) [36,38].

Only clients with valid pre- and post-CORE-OM scores and who scored above the predetermined cut-off point on the CORE-OM measure (≥10) at baseline were included in the assessment of recovery [38]. 

Regarding CORE-OM psychometric properties, the scale demonstrated good internal and test–retest reliability (0.75–0.95). The scale shows a good convergent validation against a battery of existing measures and clinician ratings of risk as well as good sensitivity to change [39].

### 2.5. Sample Size

As this was a pilot study, no sample size calculation was completed. A sample size of 180 participants was chosen for the study based on availability of existing operational resources [40].

### 2.6. Data Analysis

1. Baseline data analysis:

Analysis was conducted using SPSS Version 25 (IBM Corp, Armonk, NY, USA, 2011) [41]. Sociodemographic and clinical characteristics and baseline CORE-OM domains were analyzed based on the study dropouts (patients who dropped out from the PSW service; *n* = 30) and nondropouts (the rest of the participants; *n* = 151) using chi-square (χ^2^) and *t*-tests for categorical and continuous variables, respectively. Welch’s *t*-test was applied when equal variances were not assumed [42]. The aim was to generalize the data based on hypothetical absence of significant differences between the two groups.

2. Outcome analysis:
  - **Non-risk domains:** As the risk domain was analyzed separately, the three other domains (subjective well-being, problems/symptoms, and functioning) were analyzed to assess cluster differences among the four study arms across the four time points using means (M) and standard deviations (SD). To assess the impact of the study arms on the three non-risk CORE-OM domains, a repeated measure multivariate analysis of covariance (MANCOVA) was used for patients completing all time points (PSW: *n* = 13; TxM: *n* = 19; PSW + TxM: *n* = 13; TAU: *n* = 20). We controlled for baseline scores when analyzing across follow-up timepoints (i.e., six-weeks, three-months, and six-months) We ran the analysis with the treatment intervention (TxM yes/no and PSW yes/no) as the independent variable and mean scores of CORE-OM domains as the dependent variables while controlling for baseline scores as covariates. With regard to MANCOVA post-hoc test, Bonferroni corrections were used to control for multiple comparison error rate changes for post-hoc pairwise analyses.
  - **All-item analysis:** For CORE-OM all-item analysis (the total score of the scale), the change parameters discussed above, including CORE-OM recovery, reliable change, clinical change, and the change in prevalence, were measured. For this analysis, we compared the baseline data to six-month data only in order to examine the overall change from the beginning to the end of the intervention for the four study groups. Data are reported as proportions and percentages.
  - **Risk domain:** As the score of the risk domain correlates poorly with the nonrisk items [43], a separate analysis for risk score was carried out to examine prevalence and the associated change at the end of the study across the four groups using chi-square analysis. A client was deemed at risk if they scored ≥1 on the risk subscale.

According to the instrument manual, the CORE-OM is not limited to a particular diagnosis [44]. Thus, the tool was applied to the case mix of our study with different baseline diagnoses. The results are presented in frequencies and percentages, and a corrected two-tailed significance value of 0.05 was set as the criterion for statistical significance. Individual responses were deemed incomplete when no response was received for more than three questions (10% missing of all items); i.e., 31 questions were the minimum accepted response, with the reported items prorated to compute the means for the total scores [37]. 

## 3. Results

A total of 181 patient participants were randomized into the four arms of the study. At six weeks, 117 patients responded to the CORE-OM survey, yielding a 64.6% response rate; 103 patients responded to the survey at three months, yielding a 56.9% response rate; and 83 patients responded to the survey at six months, yielding a 45.9% response rate.

For demographic and clinical characteristics (Table 1), most participants were identified as female (56.9%), aged 25 to 34 years (28.0%), Caucasian (69.1%), with postsecondary education level (55.9%), unemployed (69.4%), single (46.9%), and admitted for depression and/or anxiety (51.1%).

Chi-square analysis indicated that the participants did not significantly differ for their sociodemographic characteristics based on the dropout condition (chi-square ranged between 0.26 and 4.66, *p* = 0.10 to 0.77). 

With respect to CORE-OM domains, the mean scores were above 1 (equivalent to above 10 on the clinical scoring) for all domains and items; *t*-test indicated no significant differences in baseline CORE-OM scores between dropout and nondropout participants (*t* value ranged between 0.02 and 1.75, *p* = 0.12 to *p* = 0.98), Table 1.

**Missing data:** One participant provided fewer than 31 CORE-OM responses and was excluded from the analysis, while five participants provided missing responses (≤2) and were included in the analysis, yielding a total of 180 eligible patients for the CORE-OM scale analysis.

The Supplementary Materials present the mean and standard deviation of CORE-OM clinical scores by study condition for the overall patient sample (A) and for those who completed assessments at each time point and at the four time points (B). None of the four groups achieved a nonclinical level (<10) on the mean scores of CORE-OM domains at either six weeks or three months. The combined group (PSW + TxM) was the only group that consistently reached a nonclinical level after six months of intervention on all nonrisk scale domains. The combined group also achieved improvement with respect to reliable change (≥5 points difference) on all nonrisk domains. 

### 3.1. Nonrisk Domains

**Figure 2:**
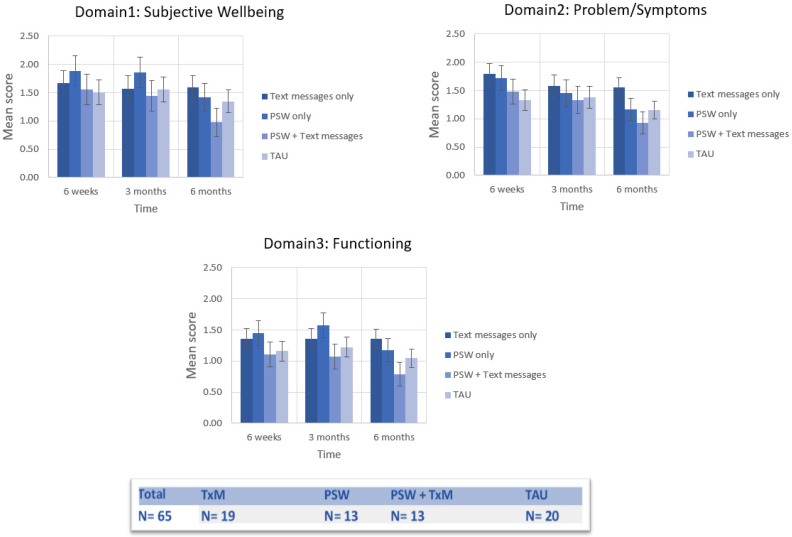
Distribution of CORE-OM domains over the six-month study period after controlling for baseline scores (adjusted means with standard error of the mean (SEM)).

Figure 2 demonstrates the distribution of CORE-OM domains over the six months of the study after controlling for the baseline scores of the domains. MANCOVA was run for participants who completed the survey at all time points (*n* = 65). With sphericity accepted for repeated measures MANCOVA, tests of within-subject effects indicated no significant effect on CORE subscores by time (*F*_6,53_ = 1.99; *p* = 0.84; ηp_2_ = 0.18), interaction of time and PSW (*F*_6,53_ = 0.62; *p* = 0.72; ηp_2_ = 0.07), and interaction of time and TxM (*F*_86,53_ = 0.17; *p* = 0.99; ηp_2_ = 0.02), or interaction of time and PSW + TxM (*F*_6,53_ = 0.36; *p* = 0.90; ηp_2_ = 0.04). By contrast, tests of between-subject effects indicated that the interaction between PSW and TxM had significant effect on the scores of CORE functioning domain (*F*_1,58_ = 4.22; *p* = 0.045; ηp_2_ = 0.07) with medium effect size.

### 3.2. All-Item Analysis

Regarding baseline and six-month data only, 82 patients completed both baseline and six-months surveys (TxM = 29 (35%), PSW = 14 (17%), PSW + TxM = 17 (21%), and TAU = 22 (27%)). With respect to the change from baseline to six months, participants who showed clinical significance for all-item score at baseline (i.e., scored above the clinical cut-off score (≥10.0)) (49, 59.8%) were deemed eligible for the analysis of clinical change and recovery parameters.

Table 2 illustrates the change parameters from baseline to six months of the CORE-OM all-item domain across the four arms of the study. 

From Table 2, the following results were reported:

1. Reliable improvement: Overall, 31/82 (37.8%) participants on the all-item score met the criteria for reliable improvement at six months. The TxM + PSW group scored the highest (58.8%).
2. Clinical change: Out of the eligible participants, 15/49 (30.6%) on the all-item score met the criteria for clinical change at six months. The TxM + PSW group scored the highest (45.5%).
3. Recovery: Out of the eligible participants, 14/49 (28.6%) met the criteria for recovery (reliable and clinically significant change) on the all-item score. Of them, 5/11 (45.5%) were from the TxM + PSW group.

It is worth mentioning that the TAU group also achieved considerable improvement on the reliable improvement (41%) and clinical change (36%) parameters, holding the second rank after the combined group (TxM + PSW).

### 3.3. Risk Subscore Prevalence Analysis

Table 3 demonstrates the prevalence of risk across the four arms of the study. The highest baseline prevalence of risk was found among the TxM + PSW group at 72.2%. After six months, the highest change (improvement) was reported for the TxM + PSW group at 50% reduction in prevalence (χ^2^_(1)_ = 9.03, *p* < 0.01), followed by the TxM group at 27.59% (χ^2^_(1)_ = 4.42, *p* = 0.04). 

## 4. Discussion

This study aimed to measure changes in psychological distress among patients with mental health disorders who were recently discharged from acute care units in Edmonton, Canada. The CORE-OM was employed to measure changes over six months of exposure to TxM and PSW interventions through a controlled observational design that included four study arms: TxM only, PSW only, TxM + PSW, and TAU arms. The TxM + PSW group showed better improvement and clinical change on the CORE-OM domains compared to the other groups. 

Generally, there was no significant difference between dropouts and the rest of the participants in the study regarding sociodemographic and clinical characteristics or the CORE-OM domains. This may indicate the potential generalizability of our data. Compared to the number of patients who completed CORE-OM at baseline, the number of patients who completed pre and post data at the three follow-up periods were relatively small (65/181 = 35.91%). Similar or lower results were reported previously, particularly in studies examining reliable responses over several time points [36,38,45]. Our data yielded no significant differences in the CORE-OM total or subscores across the study arms at baseline in terms of sociodemographic and clinical characteristics. A balanced representation was noted in biological sex distribution, which is quite a unique finding as females usually constitute a majority in similar studies [38,45]. The majority of participants were Caucasian, with postsecondary education level, unemployed, and single. 

Depression and/or anxiety disorder represented the most prevalent conditions in our study. Major depressive disorder (MDD) is the most common mental health condition, constituting a major public health problem. In 2019, it was estimated that 322 million people suffered from MDD, and it has been recognized as the single largest global contributor to nonfatal health loss, with the equivalent of over 50 million work years lost to that disability [46]. 

### 4.1. Scale Outcome across the Intervention Groups

The TxM + PSW group was the only group to achieve clinical improvement in all the CORE-OM subscores at the end of the intervention period. When we ran the analysis over the six-month period and controlled for baseline scores, combined services showed a significant association, namely with the change in the functional domain, with a medium effect size. Other studies have reported a medium to high effect size of similar interventions, such as different psychological therapies delivered in psychiatric routine care in Sweden [45], short-term counselling in Ireland [38], and person-centered care in primary care settings [47]. The comparison here, however, might not be similar given the effect sizes in the literature were reported in a pre–post research design rather than in a controlled observational model as in our study.

In our study, the TxM + PSW group was the major contributor to recovery (45.5%). This concurs with results of other studies examining similar services in the field of mental health, such as providing counselling by a therapist (46.9%) and the primary care Increasing Access to Psychological Therapy Service (50.8%). Considering the effects of diverse mental health therapies, the reported recovery rates range from 19 to 65% [38,48]. 

In this context, the proportion of the three change measures (reliable improvement, clinical change, and recovery) from baseline to six months were highest in the combined group. Peer support service combined with TxM resulted in a synergistic effect on all measures compared to either service alone or the TAU group. Recovery in our study was similar to that observed in a Swedish study, where around half of the participants reported a significant change after receiving psychiatric services [45]. This is consistent with the effectiveness of the combined service in our study. 

Our provided service included TxM, which is known to produce successful outcomes in terms of improved clinical condition and clinical measures in patients with MDD [49], eating disorder [50], anxiety disorder [26], schizophrenia-related symptoms [51], and psychiatric comorbidities [52,53]. Such web-based services have been used widely during the COVID-19 pandemic, either to monitor symptoms related to infection and direct the public to the available service channels [54] or to support mental well-being and ameliorate pandemic-related psychological distress (as in the case of the Text4Hope service) [55]. With Text4Hope, the authors provided a daily supportive text message for the public for three consecutive months. The service is effective in reducing measures of depression, anxiety, stress, and self-harm [56,57].

The combined service in our study included PSW. Peer support has been defined as “the help and support that people with lived experience of a mental illness or a learning disability can give to one another” [58]. The service has proven a success in the field of mental health, with consequent reported growth in sense of empowerment, recovery, goal orientation, and self-confidence [20,59,60,61,62]. There is an evidence-based consensus on the benefits of PSW, particularly for improved well-being [62,63,64]. In addition, PSW service integrates positive economic impacts regarding recently discharged patients in terms of reducing length of stay and readmission rate [27,65,66,67]. 

Although our study did not show a significant effect of the interventions on the problem/symptom subscale, the combined intervention was effective in improving functioning after six months. Given that the physical health and life expectancy of patients with mental illness is often poorer than the general population [68], it is notable that patients who received PSW service increasingly reported better physical health, reduced levels of distress, and better functioning [69,70]. Fortunately, a number of initiatives, such as HARP and Reclaiming Joy, emphasize achieving better quality of life and functioning in marginalized populations and patients with severe mental illness [60,71,72]. In an RCT, PSW service was incorporated into a transitional discharge model along with inpatient and community staff support [73], and the results were promising in terms of improved functionality, quality of life, and readmission rate. This again highlights synergistic effects that may result from a combined service approach in the mental health field, with expected improvement in patients’ quality of life, functioning, and hence overall productivity.

### 4.2. Risk Score

Our study showed a significant reduction in risk prevalence after six months in two main arms, namely TxM and TxM + PSW arms, which resulted in up to 50% reduction in prevalence. In a comparative study, the authors measured the risk score before and after the introduction of a counselling program to university students [36]. The reported results were promising, with a 22% reduction in risk prevalence. In another study, people who received daily supportive text messages for three months (Text4Hope) were less likely to report thoughts of self-harm and suicidal ideation during the COVID-19 pandemic compared to another group who were yet to receive the texts (odd’s ratio = 0.59) [57]. This clearly highlights the potential impact of the provided intervention in reducing risk of self-harm or other harm among those who are recently discharged from acute psychiatric care.

Our study is not without limitations. Firstly, the sample size was relatively small, particularly after dividing into four study arms. This indicates a need for replication with a larger study. Secondly, COVID-19 hit during our data collection time, so mental well-being and the provided responses of our participants may have been impacted. Thirdly, our measures were self-reported by the participants and not supported by clinical judgement or professional mental health assessment. Finally, the high dropout and/or nonservice provision rates for PSW among the study participants undermined the initial RCT design, forcing us to adopt a controlled observational study with a to-treat analysis rather than the original RCT-based intention-to-treat analysis.

While it was beyond the scope of this study, based on the results of this research, the application of novel interventions seems to be feasible as well as effective. The clinical and economic implications of incorporating PSW and TxM support into routine mental healthcare system could lead to reductions in the rates of readmission and frequent emergency visits. Currently, we have a procedure in place that examines the cost-effectiveness of this initiative moving forward. In this context, the research team has just launched a new research project that involves patients discharged from all acute care units in three of the five health care zones in Alberta [74]. This new mega study aims to examine the feasibility along with the economic impact of incorporating PSW and TxM as part of the routine health services provided to the majority of patients who are discharged from acute psychiatric care in Alberta.

## 5. Conclusions

Our study demonstrated that the provision of peer support service plus supportive text messages for six months synergistically contributes to better recovery, increased functioning, and reduction of risk symptoms for patients recently discharged from acute mental health units. Large studies with a rigorous methodology and RCT design are encouraged for future validation of our results. 

## Figures and Tables

**Table 1:** Comparison between dropout and nondropout participants regarding baseline demographic and clinical characteristics and baseline CORE-OM domain scores.

Baseline Characteristics *n* (%)	Nondropout	Dropout	χ^2^/*t* Value	*p*
	*n* = 151	*n* = 30		
**Age (years)**	41.37	41.19	*t*_(179)_ = 0.07	0.94
**Sex recorded at birth**			χ^2^_(1)_ = 0.70	0.4
Male	63 (80.8)	15 (19.2)		
Female	88 (85.4)	15 (14.6)		
**Ethnicity**			χ^2^_(2)_ = 4.66	0.1
Indigenous	18 (72.0)	7 (28.0)		
European/Caucasian	104 (83.2)	21 (16.8)		
Other	29 (93.5)	2 (6.5)		
**Educational Level**			χ^2^_(2)_ = 2.96	0.23
Less than high school	26 (89.7)	3 (10.3)		
High school degree or equivalent	38 (76.0)	12 (24.0)		
Above high school education	85 (85.0)	15 (15.0)		
**Employment Status**			χ^2^_(1)_ = 0.26	0.61
Employed	47 (85.5)	8 (14.5)		
Unemployed	103 (82.4)	22 (17.6)		
**Relationship**			χ^2^_(2)_ = 0.94	0.62
Married/common law/in relationships	42 (87.5)	6 (12.5)		
Single	68 (81.0)	16 (19.0)		
Divorced/separated/widowed	39 (83.0)	8 (17.0)		
**Admitting Diagnosis**			χ^2^_(2)_ = 0.51	0.77
Depression/anxiety	75 (81.5)	17 (18.5)		
Bipolar disorder	45 (84.9)	8 (15.1)		
Psychotic disorder	31 (86.1)	5 (13.9)		
**CORE-OM Domains (Mean Score, SD)**	*n* = 150	*n* = 30		
Subjective well-being domain	1.71 (1.02)	1.83 (1.10)	*t*_(178)_ = 0.58	0.56
Problem/symptom domain	1.75 (0.94)	1.75 (1.09)	*t*_(178)_ = 0.02	0.98
Functioning domain	1.38 (0.71)	1.66 (0.91)	*t*_(36.42)_ = 1.57	0.12
Risk domain	0.53 (0.68)	0.57 (0.66)	*t*_(178)_ = 0.29	0.77
All items	1.40 (0.73)	1.52 (0.88)	*t*_(37.45)_ = 0.68	0.5
Nonrisk items	1.59 (0.78)	1.72 (0.97)	*t*_(36.90)_ = 0.71	0.48

SD: standard deviation.

**Table 2:** The change parameters of CORE-OM all-item analysis across the four study arms from baseline to six months.

Study Groups	Prevalence *n*/*N* (%)				
	Reliable Change (Total = 82)			Clinical Change (Improvement) (Total = 49)	Recovery (Total = 49)
	Improvement	No Change	Deterioration		
**TxM**	7/29 (24.1%)	15/29 (51.7)	7/29 (24.1)	3/18 (16.7%)	3/18 (16.7%)
**PSW**	5/14 (35.7%)	7/14 (50.0)	2/14 (14.3)	2/6 (33.3%)	2/6 (33.3%)
**TxM + PSW**	10/17 (58.8%)	7/17 (41.2)	0/17 (0)	5/11 (45.5%)	5/11 (45.5%)
**TAU**	9/22 (40.9%)	12/22 (54.5)	1/22 (4.5)	5/14 (35.7%)	4/14 (28.6%)

**Table 3:** Prevalence change of risk scores across the four study arms from baseline to six months.

Condition	Prevalence, *n*/Total Responses (%)		Change in Prevalence Rate (Sixth Month from Baseline) %	χ^2^ (df)	*p* Value
	Baseline	Sixth Month			
**TxM**	18/29 (62.07)	10/29 (34.48)	−27.59	4.42 (1)	0.04 *
**PSW**	7/14 (50.00)	4/14 (28.57)	−21.43	1.35 (1)	0.25
**TxM + PSW**	13/18 (72.2)	4/18 (22.2)	−50.00	9.03 (1)	<0.01 *
**TAU**	14/22 (63.64)	12/22 (54.55)	−9.09	0.38 (1)	0.54

* *p* ≤ 0.05.

## Data Availability

The datasets used and/or analyzed during the current study are available from the corresponding author on reasonable request.

## Supplementary Materials

The following are available online at https://www.mdpi.com/article/10.3390/ijerph19073798/s1, File S1: Clinical scores of CORE-OM items across the study conditions.
