# Supplementary material for: Clinical Outcomes in Routine Evaluation Measures for Patients Discharged from Acute Psychiatric Care: Four-Arm Peer and Text Messaging Support Controlled Observational Study
Source: Int J Environ Res Public Health. 2022 Mar 23;19(7):3798. doi: 10.3390/ijerph19073798 (PMC8997547; doi:10.3390/ijerph19073798)
Supplement: Supplementary file 1 [file ijerph-19-03798-s001.zip › ijerph-1602412-supplementary.pdf]

Supplement File S1: Clinical scores of CORE-OM items across the study conditions.

A. Mean and Standard Deviation of CORE-OM clinical score by study conditions for patients who have completed the assessments at any of the four time points.

		Baseline		Six-weeks		Three-months		Six-months	
	TxM only	N= 58		N= 37		N= 33		N= 29	
	PSW only	N= 28		N= 22		N= 16		N= 14	
	PSW + TxM	N= 30		N= 26		N= 22		N= 17	
	TAU	N= 64		N= 32		N= 32		N= 22	
		Clinical Mean	SD	Clinical Mean	SD	Clinical Mean	SD	Clinical Mean	SD
Wellbeing	TxM only	18	10.14	16	10.24	17	10.63	19	11.78
	PSW only	16	11.10	17	11.27	19	10.99	13	9.65
	PSW + TxM	14	10.39	13	10.81	13	10.40	9.7	8.66
	TAU	19	9.85	17	11.38	18	11.10	15	10.62
Problem/ symptom	TxM only	17	10.42	17	8.48	17	8.04	17	10.84
	PSW only	17	8.73	15	9.58	15	9.55	11	7.97
	PSW + TxM	17	8.50	13	7.03	13	7.79	9.5	5.59
	TAU	19	9.92	15	9.11	16	11.23	14	9.77
Functioning	TxM only	15	7.61	13	8.08	15	7.78	15	9.01
	PSW only	13	7.52	14	7.18	15	6.58	11	6.38
	PSW + TxM	13	6.16	11	7.52	12	6.49	8	7.32
	TAU	15	7.86	13	8.14	13	9.00	12	7.85
Risk	TxM only	6	6.83	3	5.73	3	5.29	3	6.34
	PSW only	5	6.78	5	6.16	4	6.37	2	5.80
	PSW + TxM	4	5.77	3	5.08	1	1.72	1	2.06
	TAU	6	7.22	3	4.58	4	5.21	3	5.44
All items	TxM only	14	7.87	13	7.16	14	6.83	14	8.72
	PSW only	13	7.50	13	7.29	14	6.71	9.6	6.52
	PSW + TxM	13	6.65	11	6.17	10	6.22	8	5.35
	TAU	15	7.71	12	7.68	13	8.83	11	7.44
Non-risk items	TxM only	16	8.45	15	7.76	16	7.57	16	9.68
	PSW only	15	8.05	15	7.98	16	7.39	11	6.99
	PSW + TxM	15	7.25	12	7.01	12	7.32	9.1	6.36

TAU		17	8.34	14	8.60	15	9.78	13	8.30

B. Mean and Standard Deviation of CORE-OM clinical scores by study conditions for patients who completed assessments at the four-time points.

			Baseline		Six-weeks		Three-months		Six-months	
		N	Clinical Mean	SD	Clinical Mean	SD	Clinical Mean	SD	Clinical Mean	SD
Wellbeing	TxM only	19	14	9.06	16	10.98	15	10.18	15	11.36
	PSW only	13	14	11.16	18	11.34	18	12.08	13	9.92
	PSW + TxM	13	14	10.29	14	9.80	13	11.01	9	9.08
	TAU	20	19	10.90	17	12.08	18	11.61	15	10.98
Problem/ symptom	TxM only	19	13	11.14	17	9.64	15	8.32	14	10.72
	PSW only	13	15	8.67	17	8.70	14	10.10	11	8.30
	PSW + TxM	13	16	7.43	15	6.76	13	7.30	9	5.80
	TAU	20	18	10.94	15	9.71	15	11.64	13	10.09
Functioning	TxM only	19	12	7.27	13	8.97	13	8.11	13	8.77
	PSW only	13	11	8.84	14	5.96	15	7.21	11	6.47
	PSW + TxM	13	14	4.25	11	7.12	11	6.26	8	7.26
	TAU	20	14	7.95	13	8.98	13	9.60	12	7.97
Risk	TxM only	19	4	5.93	3	6.65	1	4.06	2	5.97
	PSW only	13	4	7.25	4	4.21	3	6.37	2	6.00
	PSW + TxM	13	4	3.94	2	3.49	1	1.58	0	0.47
	TAU	20	7	8.50	3	3.79	4	5.40	3	5.69
All items	TxM only	19	11	7.84	13	8.20	12	6.95	12	8.55
	PSW only	13	12	7.96	14	5.99	13	7.23	10	6.74

	PSW + TxM	13	13	5.27	11	5.61	10	6.10	7	5.36
	TAU	20	15	8.72	12	8.34	13	9.36	11	7.73
Non-risk items	TxM only	19	12	8.40	15	8.74	14	7.85	13	9.38
	PSW only	13	13	8.58	16	6.87	15	7.96	11	7.22
	PSW + TxM	13	15	5.89	13	6.45	12	7.23	9	6.44
	TAU	20	16	9.11	14	9.48	15	10.34	13	8.58

Clinical mean: mean x 10; W: Subjective well-being domain; P: Problems/symptoms domain; F: Functioning domain; R: Risk domain
